# Analysis of Echocardiography and Risk Factors Related to Prognosis in Adult Patients with Isolated Congenitally Corrected Transposition of the Great Arteries

**DOI:** 10.3390/jcm14155313

**Published:** 2025-07-28

**Authors:** Lixin Zhang, Yuduo Wu, Jiaoyang Xie, Yanping Ruan, Xiaoyan Hao, Hairui Wang, Ye Zhang, Jiancheng Han, Yihua He, Xiaoyan Gu

**Affiliations:** Echocardiography Medical Center, Beijing Anzhen Hospital, Capital Medical University, Beijing 100029, China; zlx7172023@163.com (L.Z.); wuyuduo123@hotmail.com (Y.W.); xiejiaoyang0330@163.com (J.X.); yanping.ruan@163.com (Y.R.); haoxiaoyan1990@163.com (X.H.); wanghairui220@126.com (H.W.); zhang33892025@163.com (Y.Z.); han_jc1977@hotmail.com (J.H.)

**Keywords:** adult congenital heart disease, isolated congenitally corrected transposition of the great arteries, tricuspid valve surgery, risk factors

## Abstract

**Objectives:** This study sought to echocardiographic manifestations and the related risk factors affecting the prognosis of isolated congenitally corrected transposition of the great arteries (CCTGA). **Methods:** A total of 143 patients (≥18 years of age) were diagnosed with isolated CCTGA at Anzhen Hospital. The patients were classified as the operation group and the non-operation group depending on whether they had undergone tricuspid valve surgery. The echocardiographic data and follow-up were compared, and the primary outcomes examined were defined as death or heart transplantation. **Results:** The average age of 143 patients with isolated CCTGA was 39.93 ± 13.50 years. Tricuspid valve surgery was performed in 31 patients with isolated CCTGA, and 112 patients did not undergo tricuspid valve surgery. The incidence of tricuspid valve structural changes in the operation group was 39.1%, and this group had higher numbers of patients with right ventricular diastolic diameter, right ventricular systolic diameter, left atrial dimensions, and regurgitation before surgery compared with the non-operation group (*p* < 0.05). Follow-up results showed no significant difference in the number of death/heart transplantations, and the incidence of systemic ventricular ejection fraction (SVEF) < 40% between the two groups. The survival rate of the surgery group was higher than that of the non-surgery group, although not statistically significant (*p* = 0.123). Age, right ventricular end-diastolic diameter, and decreased SVEF at the first diagnosis are independent predictive risk factors for major adverse outcomes. **Conclusions:** Adult patients with isolated CCTGA may have structural abnormalities in their tricuspid valves. There were no significant differences in the incidence of adverse outcomes, morphological right ventricular systolic dysfunction, and survival between the surgery group and the non-surgery group. However, this study is a retrospective study, and the sample size of the surgical group is relatively small, which may limit the generalizability of the research conclusions. In the future, a prospective, large-scale study will be conducted to evaluate the therapeutic effect of tricuspid valve surgery on such patients.

## 1. Introduction

Congenitally corrected transposition of the great arteries (CCTGA) is a rare congenital heart disease, accounting for approximately 0.05% of all congenital heart diseases [1]. CCTGA is characterized by atrioventricular (AV) discordance and ventriculoarterial (VA) discordance. More than 90% of patients are associated with other cardiac anomalies, such as ventricular septal defect (VSD), atrial septal defect (ASD), and left ventricular outflow tract obstruction (LVOT) [2]. CCTGA patients without associated cardiac anomalies are defined as isolated CCTGA with no significant hemodynamic changes. The morphological right ventricle (RV) supports the systemic circulation, and the morphological tricuspid valve is the systemic atrioventricular valve (SAVV). Patients with anatomically normal right ventricular function have good long-term survival rates [3]. However, with advancing age, these patients may develop right ventricular systolic dysfunction and increased tricuspid regurgitation, leading to congestive heart failure (CHF).

Currently, there is a lack of comprehensive studies on the survival and long-term outlook for patients with isolated CCTGA. Most of the research has focused on the prognosis of CCTGA patients with accompanying intracardiac anomalies. Some scholars have also proposed that severe SAVV regurgitation was the only independent predictor of long-term survival in CCTGA patients [4]. However, there is still no consensus on whether to perform surgery on the tricuspid valve at an early stage [5,6]. SAVV surgery may help delay the progression of anatomical right ventricular dysfunction, but it may not improve preexisting anatomical right ventricular systolic dysfunction in patients with advanced functional impairment [5]. However, there have been reports of early mortality rates of up to 25% after SAVV replacement [7]. In a study conducted by Van Son, they followed 15 CCTGA patients for up to 26 years after SAVV repair or replacement and found a survival rate of only 40% [8].

The purpose of this study was to compare the survival rates, prognosis, and echocardiograms between adult patients with isolated CCTGA who undergo SAVV surgery and those who do not. Additionally, we will investigate relevant risk factors for adverse events (death or heart transplantation) in these patients.

## 2. Materials and Methods

### 2.1. Study Population

This is a single-center, retrospective study of adults (greater than or equal to 18 years at the time of the first visit) with CCTGA that underwent transthoracic echocardiogram at Beijing Anzhen Hospital, Capital Medical University, from January 2003 to November 2023. Isolated CCTGA is defined as the absence of other cardiac malformations. We included patients diagnosed with atrioventricular conduction disorder, excluding diseases such as large ventricular septal defect and outflow tract obstruction that affect hemodynamic conditions. In our study, we did not rule out the possibility of right aortic arch disease, as relevant literature indicates that isolated right aortic arch does not affect hemodynamics.

**Figure 1:**
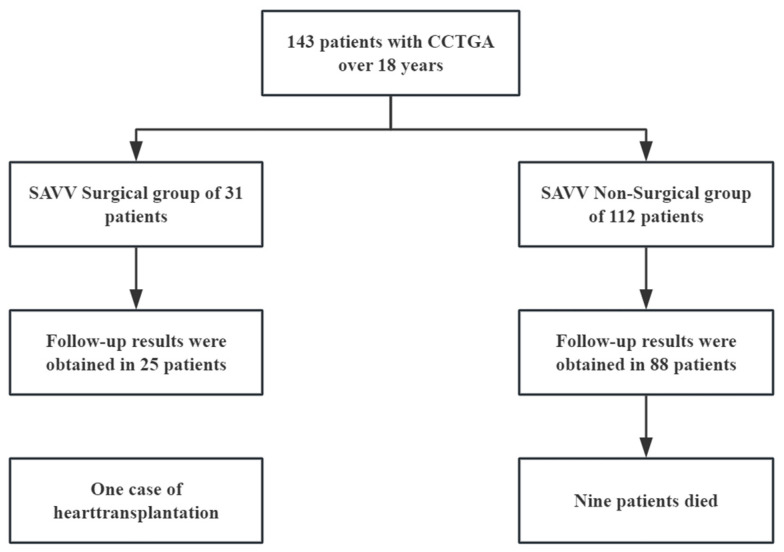
Flow chart of patient selection. CCTGA: congenitally corrected transposition of the great arteries; SAVV: systemic atrioventricular valve.

A total of 143 patients with isolated CCTGA were classified as two groups depending on whether they had undergone tricuspid valve surgery. There were 31 cases in the surgical group, among which 23 cases underwent surgical treatment at Anzhen Hospital, including 8 patients who had undergone surgery at other hospitals before follow-up at our institution, while the non-surgical group included 112 patients. The patient selection is outlined in Figure 1. The patients with follow-up information included 25 cases in the surgical group and 88 cases in the non-surgical group. The study protocol was approved by the Ethics Committee of Beijing Anzhen Hospital.

### 2.2. Clinical Data and Echocardiographic Data

Collect clinical data from patients’ initial visits, including demographic information, echocardiographic data, surgical types, and medication details. Define the echocardiogram obtained at the patient’s first visit to our hospital as the baseline data, using the baseline echocardiogram as ‘time 0’. The echocardiographic data included anatomic right ventricular end-diastolic diameter, anatomic right ventricular end-systolic diameter, atrial inner diameter, chamber wall thickness, inner diameters of the aorta and pulmonary artery and the flow velocity at the valve orifices, systemic ventricular ejection fraction (SVEF), and degree of valve regurgitation. The measurement methods of SVEF include Motion-mode (M-mode) measurement and two-dimensional measurement. According to the standards of the American Society of Echocardiography [9], SAVV regurgitation is categorized into mild, moderate, and severe level. Previous studies have defined SVEF less than 40% as indicative of anatomical right ventricular systolic dysfunction [10].

### 2.3. Tricuspid Valve Replacement/Repair

Tricuspid valve replacement (TVR): Moderate TR is an indication for TVR. If Brain Natriuretic Peptide (BNP) levels become elevated, an intervention is imminently warranted. With any TR, especially with symptoms, the patient should undergo replacement. In general, TVP was intended in all patients; however, in patients with structural leaflet malformations or severe leaflet tethering, which was noted either preoperatively or during surgery, it was decided to perform TVR.

### 2.4. Follow-Up

Follow-up data mainly included whether patients had adverse events such as admission with heart failure, status of SAVV regurgitation, size of the heart cavity, and anatomic right ventricular ejection fraction. Survival time is defined as the period from the date of the echocardiographic examination at our hospital to the last available follow-up date. Major adverse events are defined as death or heart transplantation.

### 2.5. Statistical Analysis

The continuous variables for following the Gaussian distribution were expressed as mean ± standard deviation and compared using *t* test between 2 groups, while the non-Gaussian variables were expressed as median (interquartile range (IQR)), compared using the non-parameter test between groups. The non-continuous variables were expressed as percent (%). The chi-square test or the Fisher exact test was performed to compare the variables between 2 groups. Univariable and multivariable Cox regression analysis were used to evaluate the predictors of the endpoint, and hazard ratios (HR) and 95% confidence interval (CI) were calculated. The multivariate regression analyses were performed using forward stepwise (likelihood ratio). Kaplan–Meier curves were constructed to assess the survival free from the endpoint and compared by log-rank test using SPSS Statistics software version 26.0 for Mac (IBM Corp, Armonk, NY, USA). Statistical significance was achieved with a 2-tailed value of *p* < 0.05.

## 3. Results

### 3.1. Study Population (n = 143)

The study cohort comprised 143 patients aged 18–78 years (mean 42.08 ± 13.83 years), including 69 (48.3%) women and 74 (51.7%) men. Cardiac arrhythmias were documented in 32 patients (22.4%), including atrial fibrillation in 11 cases (7.7%), supraventricular tachycardia in 6 cases (4.2%), atrioventricular block in 8 cases (5.6%), and prior pacemaker implantation in 5 cases (3.5%). Additionally, 45 patients (31.5%) received pharmacological management. Demographic characteristics are detailed in Table 1.

### 3.2. Anatomical Characteristics of the Overall Population

Among the 143 CCTGA cases, results of the cardiac positional analysis revealed levocardia in 121 (84.6%), dextrocardia in 15 (10.5%), and mesocardia in 7 (4.9%). Based on the anatomical structure, 121 cases (84.6%) were classified as situs solitus with L-looping of the ventricles and the aorta anterior and leftward of the pulmonary artery (SLL) type and 22 cases (15.4%) as situs inversus with D-looping of the ventricles and the aorta anterior and rightward (IDD) type. Concomitant cardiovascular anomalies included right aortic arch (n = 5) and persistent left superior vena cava (n = 4), as systematically summarized in Table 2.

### 3.3. Comparison of Initial Visit Echocardiographic Data Between Systemic Atrioventricular Valve Surgical and Non-Surgical Groups

In the SAVV surgical group, there were 31 cases, with 24 cases (77.4%) who underwent valve replacement and 7 cases (22.6%) who underwent valve repair. Of these, 23 cases were performed at our institution, while 8 were referred cases with postoperative follow-up data (preoperative parameters excluded from the analysis). The non-surgical group included 112 cases who did not undergo SAVV surgery. There was no significant difference in age between the two groups at their first visit. Right ventricular diastolic diameter, right ventricular systolic diameter, pulmonary artery diameter, and left atrial dimensions were all greater in the surgical group compared with the non-surgical group (*p* < 0.05). However, there was no significant difference in the SVEF. The baseline data of the two groups of patients are shown in Table 3.

The preoperative characteristics show that all 23 cases in the surgical group had severe tricuspid valve regurgitation, with 14 cases (60.9%) due to tricuspid annular dilation causing “functional regurgitation”, 2 cases (8.7%) due to tricuspid valve “leaflet dysplasia”, and 7 cases (30.4%) due to tricuspid valve “prolapse”. Notably, only one patient exhibited depressed systemic ventricular function (SVEF < 40%) with an operative age of 23 years. The mean cardiopulmonary bypass time was 146 ± 36.93 min, and the mean surgical time was 4.88 ± 1.38 h.

There were no early deaths in the surgical group. However, one patient required intra-aortic balloon pump (IABP) initiation following new-onset atrial fibrillation complicated by hemodynamic instability and severe cardiac dysfunction. Three patients (13.0%) required permanent pacemaker implantation for postoperative complete heart block. Additional complications (n = 3, 13.0%) comprised acute kidney injury, surgical site infection, and therapeutic radiofrequency ablation. Among the 5 cases that underwent repair, 1 case still had severe tricuspid regurgitation postoperatively, 3 cases had moderate regurgitation, and 1 case had mild regurgitation. In the non-surgical cohort (n = 112), severe SAVV regurgitation in 73 patients (65.2%) and impaired systemic ventricular function (SVEF < 40%) in 24 cases (21.4%) were demonstrated.

### 3.4. Comparison of Follow-Up Results Between Surgical and Non-Surgical Groups of Systemic Atrioventricular Valve

Among the patients with available follow-up data, there were 25 cases in the surgical group and 88 cases in the non-surgical group. The follow-up duration was 3.62 ± 3.70 years (minimum 0.3, maximum 20 years). The median follow-up time was 2.90 years (IQR 1.08–4.85 years). The surgical cohort demonstrated extended surveillance (6.08 ± 6.19 years, maximum 20) compared with the non-interventional group (2.92 ± 2.19 years). The average age at follow-up was 45.29 ± 11.22 years for the surgical group and 47.10 ± 14.09 years for the non-surgical group. Additionally, after discharge, 80.0% of the surgical group (n = 20) and 22.7% of the non-surgical group (n = 20) received medical treatment, including diuretics, digoxin, and beta-blockers.

There were no deaths in the surgical group. One case (4.0%) in this group underwent heart transplantation due to severe deterioration of cardiac function in the 17th year postoperatively. Comparatively, nine deaths (10.2%) occurred in the non-surgical group: heart failure-related (n = 6, 66.7%), malignant ventricular arrhythmias (n = 2, 22.2%), and infective endocarditis (n = 1, 11.1%). There was no statistically significant difference in the incidence of death/heart transplantation between the two groups (*p* = 0.570).

Postoperative echocardiographic evaluation revealed severe SAVV regurgitation in 4 patients (16.0%) undergoing tricuspid valvuloplasty procedures (TVP). The mean SVEF was 47.40 ± 11.55%. Seven patients (28.0%) demonstrated significant systolic impairment (SVEF < 40%). In the non-surgical group, severe tricuspid valve regurgitation was present in 50 patients (56.8%). The mean SVEF was 51.89 ± 9.91%, which showed no significant difference from that of the surgical group (*p* = 0.060). While 12.5% (n = 11) exhibited SVEF < 40%, this represented non-significant decline from baseline (*p* = 0.144). During the follow-up duration, the incidence of SVEF < 40% was not statistically different between the two groups. The number of people with moderate to severe tricuspid regurgitation in the non-surgical group was significantly higher than that in the surgical group, and the LA anterior–posterior diameter in the surgical group was significantly larger than that in the non-surgical group, as detailed in Table 4.

### 3.5. Survival Analysis of Surgical and Non-Surgical Groups of Systemic Atrioventricular Valve in CCTGA Patients

**Figure 2:**
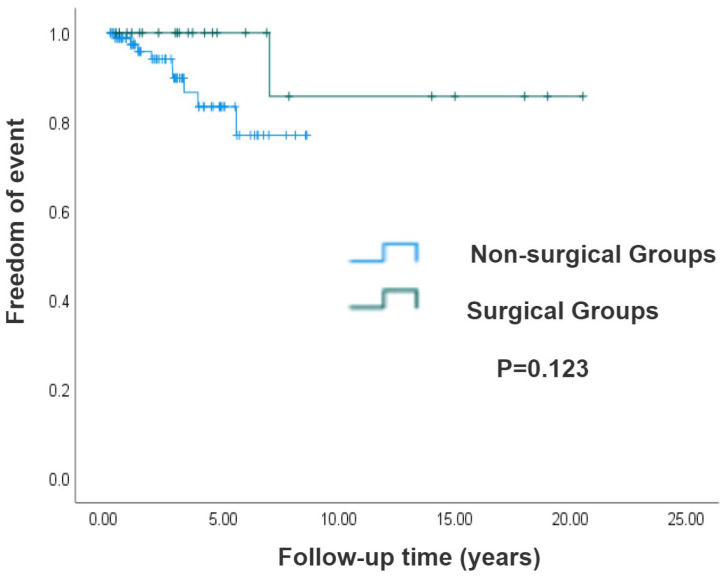
Kaplan–Meier analysis of surgical and non-surgical groups. The survival rate of the surgical group was higher than that of the non-surgical group, but the difference was not statistically significant (*p* = 0.123).

**Figure 3:**
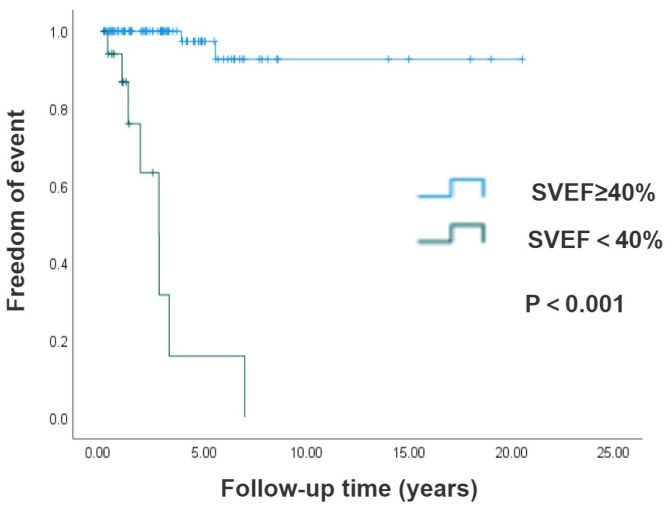
Kaplan–Meier analysis of patients with SVEF ≥ 40% and SVEF < 40%. Patients with SVEF ≥ 40% demonstrated a higher long-term survival rate, with *p* value < 0.001.

Results of the longitudinal survival analysis demonstrated superior long-term survival in the surgical cohort compared with the non-surgical group; the log-rank test showed no statistically significant difference (*p* = 0.123), as illustrated by the Kaplan–Meier survival curves in Figure 2. Moreover, we compared the long-term survival rates of 143 isolated CCTGA patients based on their SVEF being either ≥40% or <40% at the initial ultrasound examination in our hospital. Patients with preserved systolic function (SVEF ≥ 40%) demonstrated markedly superior long-term survival rates versus those with impaired ventricular performance (SVEF < 40%) (*p* < 0.001), as shown in Figure 3.

### 3.6. Risk Factors Analysis in Isolated CCTGA Patients

Univariate and multivariate Cox regression analyses were performed on 113 patients with follow-up results (25 cases in the operation group and 88 cases in the non-operation group). Results of the univariate Cox regression analysis revealed that anatomical parameters including age, the end-diastolic diameter of the right ventricle, the end-systolic diameter of the right ventricle, the end-diastolic diameter of the left ventricle, the anterior–posterior diameter of the left atrium, and the baseline SVEF value had statistical significance. Notably, the SVEF and the end-diastolic diameter of the right ventricle showed significant statistical significance (*p* < 0.001). Results of the multivariate regression analysis demonstrated SVEF as the sole independent protective factor (HR: 0.909; 95% CI: 0.851−0.971; *p*: 0.005). Age and right ventricular end-diastolic diameter emerged as independent predictors of the risk for mortality and heart transplantation. You can find the detailed results in Table 5.

## 4. Discussion

Congenitally corrected transposition of the great arteries (CCTGA) represents a rare congenital cardiac malformation characterized by heterogeneous clinical manifestations contingent upon concomitant cardiovascular anomalies. Our study was the first to investigate patients with isolated CCTGA as the research population, with the aim of analysis to eliminate the influence of other associated anomalies on prognosis. Only the natural disease course and long-term prognosis of adult patients with isolated CCTGA who underwent tricuspid valve surgery and those who did not undergo surgery were observed. The aim is to improve the prognosis consultation information for such patients in clinical practice and further enrich the understanding of the natural prognosis of this disease. Furthermore, with the continuous advancement of fetal echocardiography, this study also aims to provide prognostic consultation for the prenatal diagnosis of isolated CCTGA.

A study that examined the prognosis of 121 CCTGA patients found a 20-year survival rate of 75%, in which only 9 cases were isolated CCTGA [11]. Prieto et al. conducted a study on 40 cases of CCTGA patients, out of which 30 cases had associated intracardiac anomalies and 10 cases were isolated cases. They reported an overall 20-year survival rate of 74% [12]. In a 20-year follow-up study of 44 CCTGA patients who had not undergone cardiac surgery (22 had associated intracardiac anomalies), the Mayo Clinic discovered that poor preoperative SVEF was the sole independent predictor of the need for heart transplantation [4]. According to a recent study, SAVV surgery carries a lower risk of early mortality (0.9%) and has positive long-term outcomes, with a 20-year probability of survival or freedom from transplantation at 62.9%.

Results of the electrophysiological analysis of our cohort revealed a 22.4% incidence of clinically arrhythmias, primarily involving atrial fibrillation and high-degree atrioventricular block, consistent with previous research findings [13]. Another study on 39 CCTGA cases documented 35.9% (n = 14) atrioventricular block and 15.4% (n = 6) atrial fibrillation incidence [14]. The anomalous positioning of the atrioventricular node and abnormal atrioventricular conduction pathways in CCTGA patients predisposes them to cardiac conduction disturbances. The annual risk of developing a new atrioventricular block in CCTGA patients is approximately 2%, and atrial fibrillation may reflect a late manifestation of atrial systemic ventricular valve regurgitation [10,15]. The latest guideline (ESC 2023) emphasizes the comprehensive management of arrhythmia in patients with congenital heart disease, including the collaborative application of pacemaker implantation and drug intervention, which provides a new direction for the subsequent follow-up of this study.

The preoperative measurements of right ventricular end-diastolic diameter, right ventricular end-systolic diameter, pulmonary artery diameter, and left atrial anterior–posterior diameter were all higher in the surgical group compared with those in the non-surgical group. This is due to the severe tricuspid regurgitation in the surgical group, which leads to increased left atrioventricular volume load. However, there were no significant differences in the average age or right ventricular ejection fraction between the two groups. Follow-up showed no significant differences in adverse outcomes between the two groups. In addition to the incidence of severe tricuspid regurgitation and the difference in left anterior and posterior atrial diameter, other echocardiographic parameters, including SVEF, showed no significant differences between the two groups.

Long-term follow-up of patients with CCTGA indicates that over time [16], progressive failure of the right ventricle under systemic circulation pressure leads to tricuspid valve insufficiency [17], and 40–57% develop moderate to severe tricuspid regurgitation [18]. Among our group of 23 patients who underwent tricuspid valve replacement/repair, 39.1% had structural changes in the tricuspid valve leaflets, such as underdevelopment or prolapse. Therefore, structural abnormalities of the tricuspid valve are also the cause of tricuspid valve dysfunction. Abdelrehim et al. discovered tricuspid valve structural abnormalities in 76.9% of 80 patients, including “Ebsteinoid” malformation, underdeveloped leaflets, and prolapse [5]. A 20-year follow-up study conducted by the Mayo Clinic on 44 CCTGA patients who had not undergone prior cardiac surgery identified tricuspid valve morphological abnormalities as the primary predictor of severe systemic atrioventricular valve regurgitation [4]. This emphasizes the importance of ultrasound examiners’ need to evaluate both the severity of regurgitation and observing tricuspid valve morphology in such patients, as it is crucial for determining appropriate surgical indications.

Among the surgical group, 5 cases undergoing tricuspid valve repair showed that 4 still had moderate to severe regurgitation. It has been documented that over 50% of patients observed moderate to severe SAVV regurgitation within 20 years after repair [19], which may be caused by leaflet tethering [6]. While valve repair offers inherent advantages over replacement, there is a risk of recurrent regurgitation post-surgery, with limited therapeutic efficacy noted in treatment [20]. Among the baseline data in the non-surgical group, 65.2% had severe tricuspid regurgitation, decreasing to 56.8% during follow-up, and the incidence of SVEF < 40% decreased from 21.4% at baseline to 12.5%, potentially related to the use of inotropic and diuretic medications in patients. The data supporting drug therapy for systemic ventricular dysfunction are limited, but most experts recommend medical treatment for patients with systemic ventricular dysfunction in CCTGA [21].

There is an ongoing surgical debate about whether to replace or repair the tricuspid valve. Previous studies have indicated that the outlook for patients with isolated CCTGA significantly depends on the function of the SAVV [6,22]. SAVV regurgitation is a major factor contributing to right ventricular systolic dysfunction [6,12,22,23]. Patients without moderate to severe SAVV regurgitation have a 20-year survival rate of 93%, while those with moderate to severe regurgitation have a significantly lower rate of 49%. After surgical intervention on the SAVV, the 10-year survival rate is only 14% [12]. Some researchers believe that timely referral and tricuspid valve intervention are crucial for CCTGA patients. It can reduce the systemic ventricular load on the right ventricle and tricuspid valve, providing a better prognosis for patients with CCTGA. And it is especially applicable to those symptomatic or undergoing surgery for other cardiac conditions, as well as asymptomatic patients with severe tricuspid regurgitation or abnormal exercise test results. A study of 44 CCTGA cases with intact ventricular septum found that the SVEF of patients undergoing tricuspid valve surgery was significantly higher than that of the non-surgery group (*p* = 0.02), and the probability of avoiding death and heart transplantation was higher (*p* = 0.049) [4]. A long-term follow-up study of SAVV surgery in 108 CCTGA patients over 12.5 years found a death/heart transplantation ratio of 36.1% [5], indicating that SAVV surgery is a valuable option for treating CCTGA patients. However, the authors noted potential biases in referral and the limited sample size of these studies, which were conducted on only a subset of CCTGA patients, possibly limiting the generalizability of the results to all patients.

Viktor Hraska et al. found that TVR did not delay the deterioration of morphological right ventricle function [24]. Mongeon evaluated 46 patients, including 19 with concomitant other intracardiac anomalies; they found that for patients with preoperative SVEF < 40%, SAVV replacement did not improve SVEF, with 89% of patients still having SVEF < 40% postoperatively [25]. However, most of these studies included patients with concomitant other intracardiac anomalies; there are few studies on isolated CCTGA and a lack of relatively large-sample case studies. The prognosis of tricuspid valve surgery for patients with isolated CCTGA is still unclear.

This study shows that in the surgical group, the incidence of SVEF < 40% increased from 4.3% before the operation to 28.0%. This decline in SVEF with age suggests that the morphological right ventricle may struggle to fulfill its role in systemic circulation as individuals get older [26]. Our study did not confirm superior right ventricular function post-tricuspid valve surgery compared with the non-surgical group. This discrepancy may be related to the study’s focus on isolated CCTGA, potentially introducing bias in patient selection. For isolated CCTGA, surgical decisions need to weigh the risks and benefits more carefully. Future studies need to explore the quantitative relationship between the timing of surgery (such as the SVEF threshold) and long-term prognosis.

Our study was the first to systematically compare the long-term prognosis between the surgical and non-surgical groups in the isolated CCTGA population. Previous studies rarely compared the outcomes of isolated and non-isolated CCTGA. The multi-needle non-isolated ccTGA was the subject of the research [27,28,29]. Although not statistically significant, the long-term survival rate was higher in the surgical group compared with that in the non-surgical group. We also found that patients with an initial SVEF ≥ 40% had higher long-term survival rates compared with those with SVEF < 40%.

This study found that specific anatomical measurements, such as right ventricular end-diastolic diameter, left ventricular end-diastolic diameter, and SVEF, are linked to higher risks of adverse outcomes. Results of the Cox regression analysis showed that age was an independent risk factor for adverse events (HR = 1.103), which was consistent with the natural course of CCTGA. With the increase in age, problems such as myocardial fibrosis and conduction system disorders gradually occurred in the right ventricle. SVEF was identified as an independent predictor of mortality or cardiac transplantation in CCTGA patients. Among the adverse events observed in this group, 66.6% were linked to heart failure, while the rest were due to malignant arrhythmias and infective endocarditis. Additionally, Mongeon et al. noted that higher preoperative SVEF is associated with a better long-term prognosis [25]. The authors emphasized the importance of conducting a thorough assessment of cardiac structure and function at the initial evaluation, with particular emphasis on the right ventricular functional status.

## 5. Limitations

This study was conducted at a single-center domestic referral center for cardiac diseases, where most patients are referrals with pronounced clinical symptoms, potentially leading to selection bias. Due to the limited sample size and retrospective nature of this study, its guiding significance is limited. But the prognosis of patients after surgery might be better. Another relatively obvious limitation is that we lack long-term follow-up data and are unable to evaluate the long-term effect of the surgery. Therefore, there is a critical need for further large-scale, long-term follow-up studies to evaluate the prognosis and survival outcomes of tricuspid valve surgery in CCTGA patients and determine optimal treatment approaches.

## 6. Conclusions

In summary, isolated CCTGA patients may not show significant hemodynamic abnormalities but might have tricuspid valve structural changes requiring thorough ultrasound evaluation for surgical consideration. Our study did not find significant differences in postoperative adverse event rates, right ventricular size, average SVEF, or incidence of right ventricular systolic dysfunction between the surgical and non-surgical groups. However, the surgical group displayed a higher long-term survival rate compared with the non-surgical group, although this difference was not statistically significant. Hence, further large-scale multicenter studies are needed to establish the benefits of tricuspid valve surgery for isolated CCTGA patients. Moreover, an initial SVEF ≥ 40% at diagnosis predicts higher long-term survival rates compared with SVEF < 40%. Age, right ventricular end-diastolic diameter, and SVEF were independent predictors of death/heart transplantation in patients with CCTGA.

## Figures and Tables

**Table 1:** Baseline Data at the First Visit.

Characteristics	Values
**Total**	143
Age at initial visit (years)	42.08 ± 13.83
Sex, men	74 (51.7)
**Arrhythmia**	32 (22.4)
Pacemaker implanted	5 (3.5)
Atrial fibrillation	11 (7.7)
Supraventricular tachycardia	6 (4.2)
High-degree atrioventricular block	8 (5.6)
Premature ventricular complexes	2 (1.4)
**Medication**	45 (31.5)
ACEI/ARB	6 (4.2)
Beta-blocker	8 (5.6)
Digoxin	16 (11.1)
Diuretic	30 (20.1)
Anticoagulant	10 (7.0)

Data are expressed as mean ± SD, median with IQR, or n with percentage. ACEI, angiotensin-converting enzyme inhibitor; ARB, angiotensin receptor blocker. The medication data were only collected from patients treated in our hospital during hospitalization.

**Table 2:** Anatomical Characteristics of the Overall Population.

Characteristics	Values
**Segmental anatomy**	
SLL	121 (84.6)
IDD	22 (15.4)
**Cardiac malposition**	
Dextrocardia	15 (10.5)
Mesocardia	7 (4.9)

SLL: situs solitus with L-looping of the ventricles and the aorta anterior and leftward of the pulmonary artery; IDD: situs inversus with D-looping of the ventricles and the aorta anterior and rightward.

**Table 3:** Baseline data of the first visit in the isolated CCTGA patients’ tricuspid valve surgery group and the non-surgery groups.

	Surgical Group	Non-Surgical Group	*p* Value
Cases	23	112	
Age (years)	41.78 ± 11.56	42.72 ± 14.08	0.765
SAVV regurgitation grade			
Mild	0	39 (34.8)	
≥Moderate	23 (100)	73 (65.2)	**<0.001**
SVEF (%)	52.26 ± 10.13	50.61 ± 11.91	0.494
SVEF < 40%	1 (4.3)	24 (21.4)	
RV diastolic diameter (cm)	61.65 ± 8.51	54.78 ± 10.76	**0.008**
RV systolic diameter (cm)	46.35 ± 10.69	40.22 ± 11.31	**0.041**
LV diastolic diameter (cm)	44.60 ± 10.52	46.00 ± 12.34	0.664
LV systolic diameter (cm)	38.00 ± 4.32	28.93 ± 8.93	0.056
Pulmonary artery diameter (cm)	29.13 ± 9.42	24.72 ± 6.44	**0.007**
LA anterior–posterior diameter (cm)	51.75 ± 13.50	41.10 ± 9.27	**<0.001**
LA superior–inferior diameter (cm)	67.64 ± 20.83	58.95 ± 11.68	0.073
LA left–right diameter (cm)	58.24 ± 19.63	49.49 ± 10.42	0.061
RA superior–inferior diameter (cm)	47.82 ± 13.62	46.39 ± 9.35	0.614
RA left–right diameter (cm)	38.59 ± 13.63	38.52 ± 7.86	0.985

Data are expressed as mean ± SD, median with IQR, or with percentage. LA: left atrium; RV: right ventricular; SVEF: systemic ventricular ejection fraction.

**Table 4:** Comparison of Follow-up Results Between Surgical and Non-surgical Groups of Isolated CCTGA Patients.

	Surgical Group	Non-Surgical Group	*p* Value
Total follow-up time (years)	3.62 ± 3.70		
Follow-up time (years)	6.08 ± 6.19	2.92 ± 2.19	**0.019**
Number of patients followed	25	88	
Mild regurgitation	4 (16.0)	20 (22.7)	0.468
≥Moderate regurgitation	4 (16.0)	50 (56.8)	**<0.001**
LA anterior–posterior diameter (cm)	46.86 ± 8.34	40.93 ± 6.92	**0.001**
RV end-diastolic diameter (cm)	57.26 ± 11.25	54.59 ± 9.35	0.254
RV end-systolic diameter (cm)	40.93 ± 10.87	40.27 ± 10.28	0.832
SVEF (%)	47.40 ± 11.55	51.89 ± 9.91	0.060
SVEF < 40%	7 (28.0)	11 (12.5)	**0.119**
Death/heart transplantation	1 (4.0)	9 (10.2)	0.570

Data are expressed as mean ± SD, median with IQR, or with percentage. SVEF: systemic ventricular ejection fraction; LA: left atrium; RV: right ventricle.

**Table 5:** Univariate and Multivariate Analysis for Predictors of the Primary Outcome.

Univariate Analysis	Hazard Ratio	95% Confidence Interval	*p* Value
Age (years)	1.068	1.019–1.120	**0.006**
Women	1.472	0.228–4.566	0.548
Surgery	0.217	0.049–3.413	0.155
RV end-diastolic diameter (cm)	1.110	1.019–1.154	**<0.001**
RV end-systolic diameter (cm)	1.114	1.028–1.164	**<0.001**
LV end-diastolic diameter (cm)	1.057	1.010–1.171	**0.047**
LV end-systolic diameter (cm)	1.122	1.016–1.234	**0.021**
Pulmonary artery diameter (cm)	1.046	0.993–1.107	0.088
LA anterior–posterior diameter (cm)	1.111	1.007–1.225	**0.036**
LA superior–inferior diameter (cm)	1.023	0.966–1.083	0.435
LA left–right diameter (cm)	1.055	1.000–1.113	**0.050**
RA superior–inferior diameter (cm)	1.033	0.986–1.083	0.169
RA left–right diameter (cm)	1.028	0.970–1.089	0.353
SVEF (%)	0.866	0.808–0.928	**<0.001**
Severe regurgitation	0.017	0.000–7.874	0.194
≥Moderate regurgitation	0.033	0.000–68.304	0.382
**Multivariate analysis**			
Age (years)	1.103	1.021–1.191	**0.013**
RV end-diastolic diameter (cm)	1.116	1.018–1.224	**0.020**
SVEF (%)	0.909	0.851–0.971	**0.005**

RV: right ventricular; LV: left ventricular; LA: left atrial; RA: right atrial; SVEF: systemic ventricular ejection fraction.

## Data Availability

The data underlying this article will be shared on reasonable request to the corresponding author.
